# Comparison of the effectiveness of position change for patients with pain and vascular complications after transfemoral coronary angiography: a randomized clinical trial

**DOI:** 10.1186/s12872-021-01922-w

**Published:** 2021-02-25

**Authors:** Hojjat Niknam Sarabi, Zahra Farsi, Samantha Butler, Amir Hosein Pishgooie

**Affiliations:** 1 Student Research Committee and Military Nursing Department, Faculty of Nursing, Aja University of Medical Sciences, Tehran, Iran; 2 Research and Community Health Department, Faculty of Nursing, Aja University of Medical Sciences, Kaj St., Shariati St, Tehran, Iran; 3 Department of Psychiatry, Harvard Medical School, Children’s Hospital Boston, Boston, MA USA; 4 Medical-Surgical Department, Faculty of Nursing, Aja University of Medical Sciences, Tehran, Iran

**Keywords:** Hemorrhage, Cardiac catheterization, Coronary angiography, Hematoma, Pain

## Abstract

**Background:**

Prolonged immobilization after transfemoral coronary angiography (TFA) may cause pain and vascular complications in patients. This study aimed to evaluate the effectiveness of a change in position to decrease pain and vascular complications for patients after TFA.

**Methods:**

This randomized clinical trial was conducted in 2020. Purposive sampling of 72 eligible patients undergoing TFA were selected and randomly assigned to either an experimental or control group. Patients in the experimental group (EG) were placed in a supine position for 2 h after angiography, followed by a semi-seated position with the bed angle gradually increased to 45° over 4 h. Patients in the control group (CG) remained in the supine position for 6 h. Vital signs, groin, back and leg pain, hematoma, hemorrhage, and urinary retention were assessed in both groups before, immediately after, and over 6 h after angiography. The Visual Analogue Scale was used to measure pain, the Christensen scale to measure hematoma, counting bloody gases to measure hemorrhage, and patient self-rating to determine urinary retention.

**Results:**

There was no significant difference between EG and CG on score of groin (2.69 ± 1.00 vs. 2.61 ± 1.00, P = 0.74), back (2.19 ± 0.98 vs. 2.47 ± 0.87, P = 0.21), and leg pain (2.14 ± 0.71 vs. 2.50 ± 1.08, P = 0.27) before the TFA. However, from the second hour to the sixth hour after the TFA, the pain in the EG was significantly less than the CG (P < 0.001). So that pain in the groin (1.36 ± 0.48 vs. 3.28 ± 0.81), back (1.25 ± 0.50 vs. 3.81 ± 1.06), and leg (1.44 ± 0.55 vs. 3.28 ± 0.81) for the EG patients was significantly less than the CG in the sixth hour after TFA (P < 0.001). No patients experienced hematoma. No differences were noted between groups in hemorrhage and urinary retention.

**Conclusions:**

Position change to a semi-seated position in patients after TFA is effective and safe for reduction of pain without increasing vascular complications.

***Trial registration*:**

*Iranian Registry of Clinical Trials:* IRCT registration number: IRCT20200410047011N1, Registration date: 30/04/2020

## Background

Cardiovascular diseases (CVD) and vascular disorders account for more than 12% of all diseases around the world [1, 2]. Coronary artery disease (CAD) accounts for 20% deaths in industrialized countries [3] and 78% in developing countries [1, 2]. In Iran, CVD and CAD are common with an increasing rate of death (50% currently) and disability [4, 5].

Early and accurate diagnosis is imperative in patients with CAD. Coronary angiography is considered the gold standard for the diagnosis of significant CAD [6]. However, coronary angiography is associated with complications in approximately 0.7 to 28% of cases [7, 8]. Complications that impact mortality and treatment cost include myocardial infarction, arrhythmia, hematoma, hemorrhage, and ecchymosis [9, 10]. Complications of angiography are dependent on multiple factors including a patient’s vascular anatomy, co-morbid conditions such as history of current smoking, increased body mass index (BMI), high blood pressure, diabetes mellitus, elevated cholesterol levels, the angiography performed, and the experience of the medical team and hospital unit [8, 11, 12].

Transradial and transfemoral are the two main approaches to angiography which are used for diagnostic and therapeutic purposes in catheterization. Transfemoral coronary angiography (TFA) is often preferred over transradial due to the unlimited repetition of puncturing, easy access, less radiation, and less contrast [13]. However, TFA is associated with acute and chronic complications. For example, back pain is a common complication following TFA and is associated with immobility and restricted positioning following the procedure [14]. It is recommended that following TFA, patients complete bed rest in supine for 6–12 h to prevent possible complications [15]. This extended bed rest may lead to further patient discomfort, groin and back pain, increased treatment costs, and a longer hospital stay [16]. Other complications after TFA are hematoma, hemorrhage, and urinary retention [17, 18]. To reduce complications from TFA, strategies such as therapeutic positioning of the patient, increase the head of bed elevation [19], early ambulation [18, 19], and use of a weight applied to the catheter insertion site [19, 20] are recommended, but the effectiveness of these methods are controversial. Changing the patient’s position, increasing the head of bed elevation, and early mobilization after the angiography can reduce back pain [18, 21], groin pain, urinary retention [18], and overall increase patient comfort [21, 22] without an increase in the vascular complications such as hematoma, hemorrhage, thrombosis, or bruising [23] and decrease the healthcare providers’ workload, reduce the duration of hospitalization and also enable the patients to meet their needs such as eating, drinking, and voiding [22]. However, there is varying information in the literature on the effect of adjusting the bed angle. For example, evidence showed that slightly raising the head of bed (15°) after angiography did not reduce the pain/discomfort of patients [24]. Besides, an incline of 30° did not affect pain intensity, urinary retention, and other vascular complications after angiography compared to the supine position [25] but, an incline of 45° was found can help to decrease overall pain [26]. To now, many hospitals are requiring their patients to remain in long-time bed rest after FFA to prevent complications. Based on the evidence mentioned, there is no consensus regarding the optimal position and length of bed rest after TFA. According to contradictory evidence, the current study investigated the effect of increasing patient bed elevation and changing the patient’s position on pain and vascular complications after TFA. It was hypothesized that an increase in bed elevation and position changes would be safe and effective in reducing pain and vascular complications.

## Materials and methods

### Design

This study was planned and carried out according to CONSORT guideline for randomized clinical trials on the angiography and post-angiography wards of 502 Hospital, Tehran, Iran and registered in the Iranian Registry of Clinical Trials (No. IRCT20200410047011N1, Registration date: 30/04/2020). Eligible patients included those admitted to the cardiovascular ward between May and June, 2020 with CAD who underwent TFA. Patients were randomly assigned to either the experimental group (EG) or the control group (CG) by tossing a coin. This study was a double blinded trial. The researcher assistant enrolled patients and assigned them to interventions. Outcome evaluator, the patients, and statistical analyst were blinded to group allocation.

#### Participants and setting

**Fig. 1:**
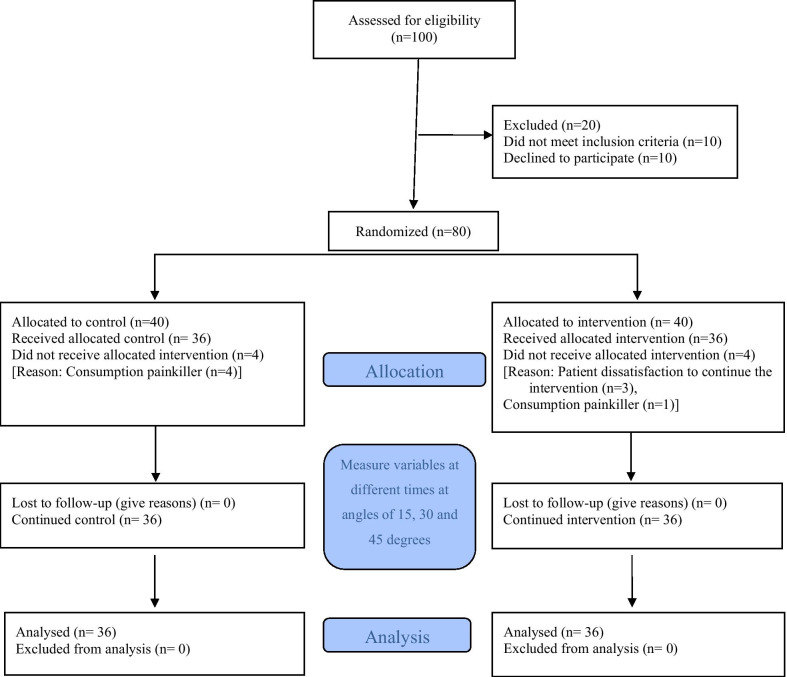
The study process

Based on a previous study [18], the appropriate sample size was estimated at 33 patients for both the EG and CG with a power of 80% and α of 0.05, using G-Power software version 3.0.10. A 10% drop-out rate was estimated. A total of 100 patients were recruited for the study and the final sample included 72 patients by purposive sampling method (see CONSORT flow diagram in Fig. 1).

The inclusion criteria encompassed: (1) willingness to participate in the study, (2) conscious, (3) lack of hemophilia and other coagulation disorders, (4) normal prothrombin time (PT) and international normalized ratio (INR) tests, (5) candidate for TFA, (6) no past treatment with streptokinase, (7) no history of previous low back pain, (8) no history of severe hemorrhage, extensive hematoma or pain intensity greater than 4.5 based on the Visual Analog Scale (VAS), and (9) no history of analgesic or opioid use before, during, and after TFA. Exclusion criteria included: (1) withdrawal to continue in the study and (2) worsening of the patient's condition such as pain more than 7, active hemorrhage, and unstable vital signs. In this study, all patients used tablet aspirin 80 mg, Plavix 300 mg single dose, ampule diazepam 5 mg, and ampule promethazine 25 mg for prophylaxis according to the physician order before TFA.

### Measures

Demographic and medical history data were collected from the patients’ medical records. Individual characteristics included age, sex, and previous history of angiography, and history of cardiac surgery.

Primary outcomes including pain scores, size of hematoma, and amount of hemorrhage were collected eight times in 1-h intervals starting at 1 h before the TFA, immediately after the TFA, and every hour for the next 6 h following the TFA. Urinary retention was assessed before, during, and 10 min after the TFA.

Secondary outcomes including vital signs such as pulse rate, respiratory rate, body temperature, and systolic and diastolic blood pressure were recorded throughout the study, starting at 1 h before and up to 6 h after TFA. Vital signs were measured using telemetry based vital sign monitoring.

Pain was measured using the VAS, a subjective, validated measure to assess acute and chronic pain with high reliability [27]. The VAS asked participants to create a handwritten mark on a 10-cm line that represents a continuum of pain where scores range from zero to ten. Scores are divided into pain categories (0–1: no pain, 2–3: low pain, 5–4: high pain, 7–6: very high pain, 9—8: maximum pain, 10 indescribable pain) [28, 29]. Pain scores were recorded for the groin, back, and leg.

Hematoma was measured using the Christensen scale. On the Christensen scale, a small hematoma was noted as measuring between 2 and 5 cm^2^ and a large hematoma was equal to or greater than 5 cm^2^. In an irregularly shaped hematoma, the largest and smallest diameters of the hematoma are measured and the area of the hematoma is calculated by multiplying the diameters by each other [30]. Cohen's Kappa coefficient was 0.94 that showed the inter-rater reliability for the Christensen scale. The amount of blood loss was calculated based on the number of bloody gases collected. The amount of blood lost was calculated in ml. The amount of hemorrhage in each bloody gas (4 × 4 cm) was considered to be about 10 ml. The number of bloody gases was multiplied by 10 ml [31]. The inter-rater reliability for the amount of blood loss was confirmed by Cohen's Kappa coefficient that was 0.90. The urinary retention was collected through self-report from the patient (yes/no).

All variables were collected by the researchers. Quality assurance and validity of data collection were provided by 12 nursing professors from Aja, Tehran, Mashhad, and Tarbiat Modares University of Medical Sciences.

All the beds used in this study were electric intensive care unit (ICU)/cardiac care unit (CCU) beds and the angle of the bed could be adjusted easily to the precise angle required of the study protocol. Beds were equipped with four motors, resistant to water and dust penetration, and were adjustable by four actuators without disturbing the patient.

### Intervention

Patients in the CG received the standard care provided at the study institution following TFA which included positioning in supine for 6 h after the TFA and the affected leg was straight and immobilized. Patients were asked not to move their legs during this time and a sandbag weighing about 4 kg [21, 32] was placed on the catheter dressing for 6 h to prevent vascular complications. Patients were required to remain supine with only small movements. After this initial 6 h of limited movement, the patients completed another 4–6 h of bed rest where they could move freely while in the bed. After these 10–12 h in the bed, patients were recommended to get out of bed and if there were no complications, they were discharged the day after the TFA.

All interventions in the EG were performed by one of the researchers, under the supervision of a physician, who was a master’s student of emergency nursing with 19 years of experience in ICU care. Patients in the EG were kept in the supine position for 2 h after angiography. The affected leg was straight and immobile. After 2 h of immobility, the EG patient was transitioned to a semi-seated position. Over the next 4 h, the bed angle gradually increased from 0° to 45°. For example, in the second, third, and fourth hour, the bed was placed at an angle of 15°, 30°, 45°, respectively. It should be noted that in all stages of the intervention for the EG, a sandbag was located on the dressing area of the arterial access and the patients' leg was straight. From the third hour onwards, a small pillow was placed under the patients' waist area for comfort. Throughout the intervention in the EG, specialist physicians and experienced nurses were available in case of serious complications such as active hemorrhage, severe pain, or patient dissatisfaction.

### Data analysis

Data were analyzed using SPSS software version 22 (SPSS, Inc. Chicago, IL, USA) and descriptive and analytical tests. Kolmogorov–Smirnov test was used to check the normality of the data. The homogeneity of groups for demographic and clinical characteristics of the patients was assessed using independent sample t-test and Fisher's exact test. Also, homogeneity of groups for baseline condition was investigated by the independent sample t-test. To compare the mean changes of the outcome variables immediately after TFA, 1st, 2nd, 3rd, 4th, 5th, and 6th hours after TFA compared to the baseline between groups, the researchers employed repeated measures analysis of variance (RM-ANOVA). The significance level was considered less than 0.05.

## Results

### Characteristics of patients

The mean age of patients was 62.21 ± 12.22 (range 21–88) years. Forty-two (58.3%) of the patients were male, 64 (88.9%) patients had no incidence of cardiac surgery, and 43 (59.7%) patients had previous angiography experience. There was no significant difference between the two groups in terms of any of the individual baseline characteristics (P < 0.05). See Table 1.

**Table 1:** Individual characteristics and medical background of the patients in the CG and EG

Variable	Groups		P value
	Experimental (n = 36) Mean ± SD or n (%)	Control (n = 36) Mean ± SD or n (%)	
Age, mean ± SD, year	63.44 ± 9.87	60.97 ± 14.23	0.39^a^
Sex			
Male	22(68.0)	20(55.0)	0.63^b^
Female	14(32.0)	16(45.0)	
Previous cardiac surgery			
Yes	6(17.0)	2(6.0)	0.67^b^
No	30(87.0)	34(94.0)	
Previous coronary angiography			
Yes	24(66.0)	20(55.0)	0.46^b^
No	12(44.0)	16(45.0)	

Abbreviations: SD, Standard Deviation; n, Number

^a^Independent t test

^b^Fisher exact’ test

### Primary outcomes

**Fig. 2:**
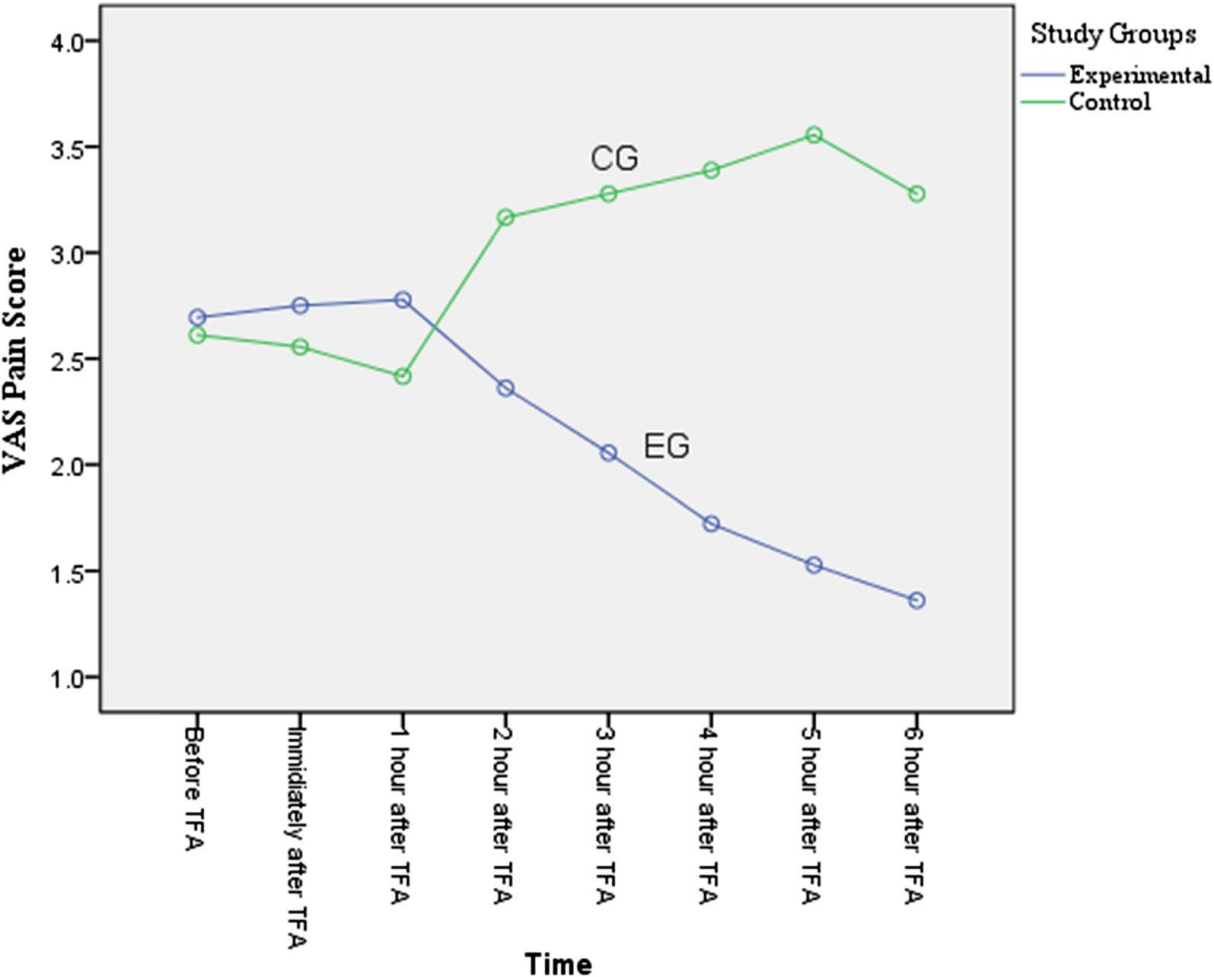
Groin pain scores for EG verse CG

**Fig. 3:**
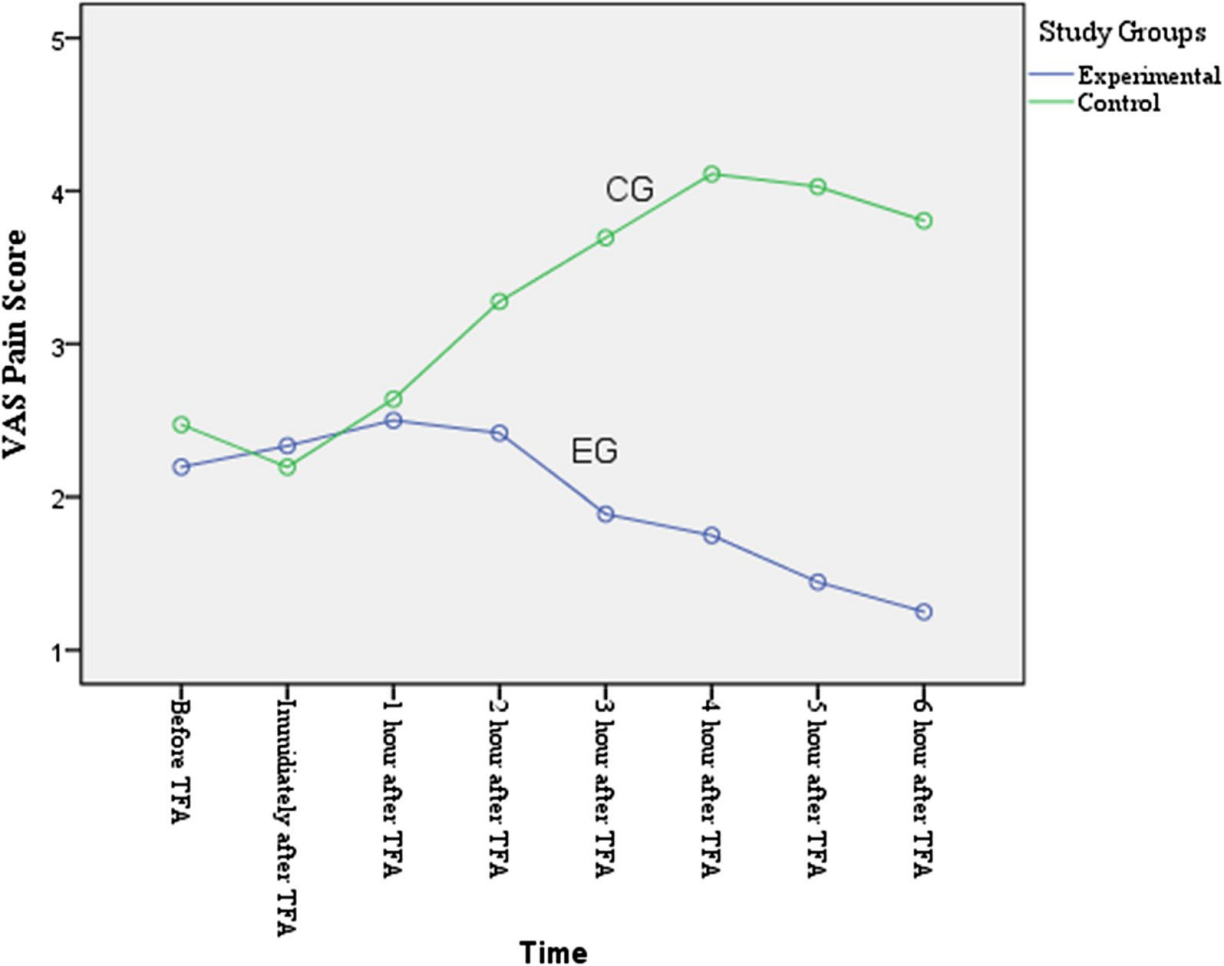
Low back pain scores for EG verse CG

**Fig. 4:**
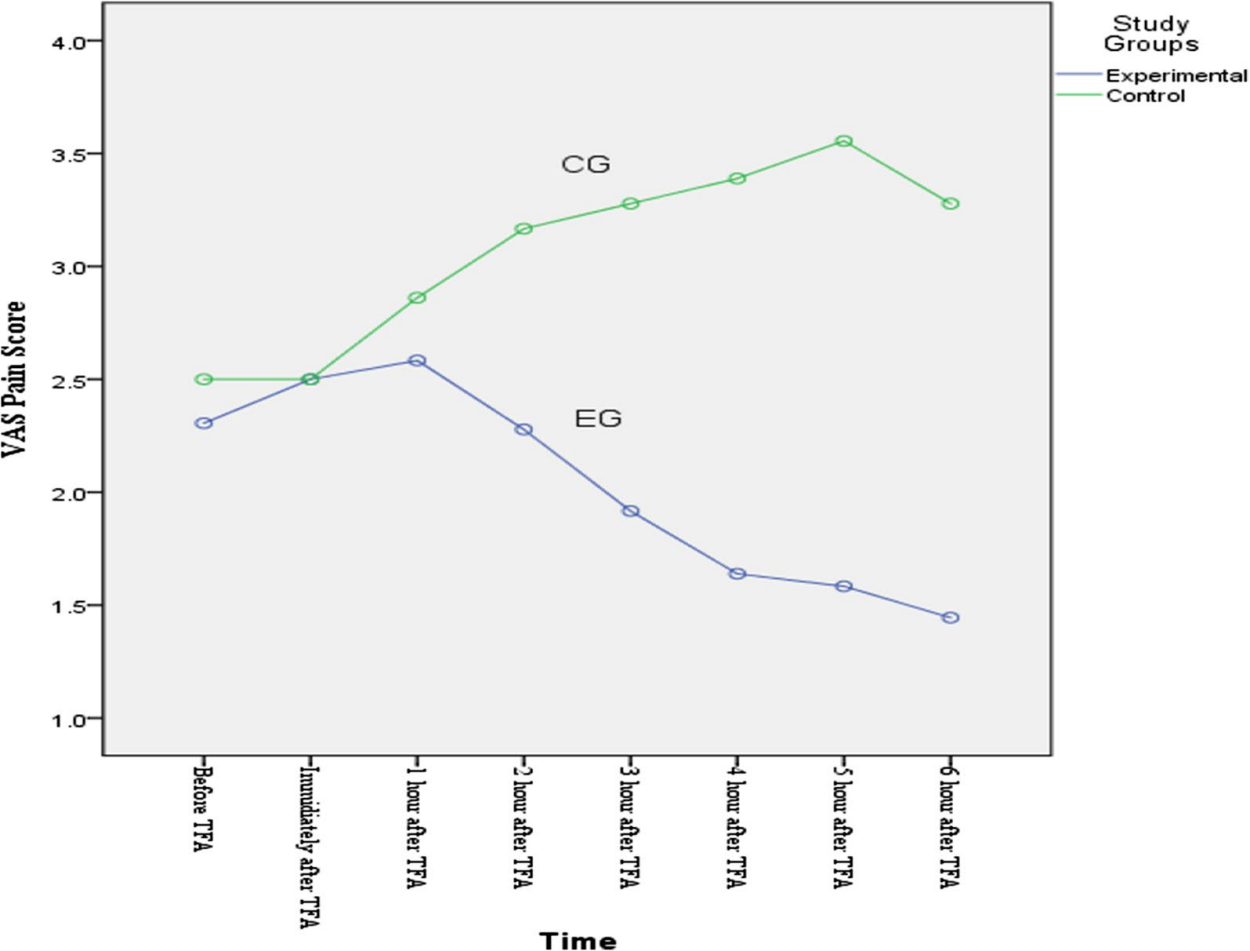
Leg pain scores for EG verse CG

None of the patients had severe pain and/or hemorrhage. There was no significant difference between groin (P = 0.74), back (P = 0.21), and leg pain (P = 0.27) in the two study groups before the TFA. However, from the second hour to the sixth hour after the TFA, the pain in the EG was significantly less than the CG (P < 0.001 for each hour segment). See Table 2. RM-ANOVA showed a decreasing trend of the groin, leg, and back pain in the EG in the second to sixth hours after the trial (P < 0.001 for each area of pain measured), while in the CG groin, leg and back pain had an increasing trend (P < 0.001 for each area of pain measured) (Fig. 2, 3, 4).

**Table 2:** Comparison of changes in patients' pain scores by study groups

Variable	Time	Study Group		P value
		Experimental (n = 36) Mean ± SD	Control (n = 36) Mean ± SD	
Groin pain measurement	Before TFA	2.69 ± 1.00	2.61 ± 1.00	0.74^a^
	Immediately after TFA	2.75 ± 0.80	2.56 ± 0.80	0.31^a^
	1st hour after TFA	2.78 ± 0.98	2.42 ± 1.02	0.13^a^
	2nd hour after TFA	2.36 ± 0.79	3.17 ± 0.69	< 0.001^a^
	3rd hour after TFA	2.06 ± 0.67	3.28 ± 0.74	< 0.001^a^
	4th hour after TFA	1.72 ± 0.56	3.39 ± 0.79	< 0.001^a^
	5th hour after TFA	1.53 ± 0.60	3.56 ± 0.77	< 0.001^a^
	6th hour after TFA	1.36 ± 0.48	3.28 ± 0.81	< 0.001^a^
Between group (F = 71.53, P < 0.001) ^b^		F = 29.53, P < 0.001^b^	F = 12.95, P < 0.001^b^	
Back pain measurement	Before TFA	2.19 ± 0.98	2.47 ± 0.87	0.21^a^
	Immediately after TFA	2.33 ± 0.86	2.19 ± 0.62	0.43^a^
	1st hour after TFA	2.50 ± 0.91	2.64 ± 1.07	0.55^a^
	2nd hour after TFA	2.42 ± 0.87	3.28 ± 0.70	< 0.001^a^
	3rd hour after TFA	1.89 ± 0.70	3.68 ± 0.78	< 0.001^a^
	4th hour after TFA	1.75 ± 0.64	4.11 ± 0.85	< 0.001^a^
	5th hour after TFA	1.44 ± 0.55	4.03 ± 0.91	< 0.001^a^
	6th hour after TFA	1.25 ± 0.50	3.81 ± 1.06	< 0.001^a^
Between group (F = 45.127, P < 0.001)^b^		F = 20.98, P < 0.001^b^	F = 31.89, P < 0.001^b^	
Leg pain measurement	Before TFA	2.14 ± 0.71	2.50 ± 1.08	0.370^a^
	Immediately after TFA	2.50 ± 0.77	2.50 ± 0.84	1.00^a^
	1st hour after TFA	2.58 ± 0.90	2.86 ± 1.17	0.26^a^
	2nd hour after TFA	2.28 ± 0.74	3.17 ± 0.69	< 0.001^a^
	3rd hour after TFA	1.92 ± 0.55	3.28 ± 0.74	< 0.001^a^
	4th hour after TFA	1.64 ± 0.68	3.39 ± 0.76	< 0.001^a^
	5th hour after TFA	1.58 ± 0.55	3.56 ± 0.77	< 0.001^a^
	6th hour after TFA	1.44 ± 0.55	3.28 ± 0.81	< 0.001^a^
Between group (F = 84.50, P < 0.001)^b^		F = 20.41, P < 0.001^b^	F = 10.28, P < 0.001^b^	

*SD* standard deviation, *TFA* transfemoral coronary angiography

^a^Independent t test

^b^RM-ANOVA

Before and after the TFA there was no indication of hematoma for any of the patients in either the EG or CG. Fisher's exact test showed that patients in both groups had no significant hemorrhage before (P = 0.18), immediately after (P = 0.08), in the first hour (P = 0.62), or any time up to the sixth hour after TFA (P = 0.90). After the TFA, patients in two groups did not experience significant hemorrhage. Fisher's exact test showed that none of the patients complained of urinary retention before the TFA and urinary retention in patients immediately, ten minutes after TFA was not statistically different (P > 0.05).

### Secondary outcomes

There was no significant difference between the mean pulse rate, respiration rate, body temperature, or systolic and diastolic blood pressure in the two groups before the TFA. But the mean respiratory rate of the patients in the two groups immediately after TFA (P = 0.042) and 1 h after TFA (P = 0.021) was significantly different with the EG showing the most optimal respiratory rate. There was a significant difference in body temperature in the 6th and final hour after the trial (P = 0.018). The RM-ANOVA test showed an almost constant trend of pulse rate, respiratory rate, body temperature, and systolic and diastolic blood pressure in the EG and CG in all hours before and after TFA (P > 0.05). See Table 3.

**Table 3:** Comparison of changes in patients' vital signs by study groups

Variable	Time	Study group		P value
		Experimental (n = 36) Mean ± SD	Control (n = 36) Mean ± SD	
Pulse rate, BPM	Before TFA	76.64 ± 10.59	75.50 ± 10.35	0.955^a^
	Immediately after TFA	76.31 ± 7.5	78.83 ± 10.03	0.241^a^
	1st hour after TFA	75.33 ± 10.53	76.19 ± 10.60	0.731^a^
	2nd hour after TFA	75.23 ± 14.23	75.69 ± 10.45	0.876^a^
	3rd hour after TFA	76.42 ± 6.86	75.50 ± 10.35	0.659^a^
	4th hour after TFA	77.69 ± 6.64	76.19 ± 10.60	0.955^a^
	5th hour after TFA	76.58 ± 7.65	74.03 ± 10.26	0.235^a^
	6th hour after TFA	76.14 ± 6.87	76.36 ± 10.55	0.916^a^
Between group (F = 0.103, P = 0.749)^b^		F = 0.42, P = 0.7^b^	F = 4.15, P = 0.1^b^	
Respiration rate, bpm	Before TFA	18.30 ± 2.40	19.30 ± 1.77	0.55^a^
	Immediately after TFA	18.31 ± 2.13	18.25 ± 1.77	0.042^a^
	1st hour after TFA	18.39 ± 1.47	19.31 ± 1.80	0.021^a^
	2nd hour after TFA	19.31 ± 1.21	19.56 ± 1.15	0.374^a^
	3rd hour after TFA	18.83 ± 0.94	19.36 ± 1.77	0.120^a^
	4th hour after TFA	18.67 ± 2.30	19.36 ± 1.77	0.157^a^
	5th hour after TFA	19.72 ± 2.27	19.31 ± 1.80	0.392^a^
	6th hour after TFA	18.56 ± 1.42	19.56 ± 1.57	1^a^
Between group (F = 2.492, P = 0.119)^b^		F = 3.96, P = 0.07^b^	F = 0.87, P = 0.41^b^	
Body temperature, ºC	Before TFA	36.31 ± 0.46	36.50 ± 0.50	0.095^a^
	Immediately after TFA	36.47 ± 0.50	36.42 ± 0.50	0.641^a^
	1st hour after TFA	36.42 ± 0.50	36.36 ± 0.48	0.634^a^
	2nd hour after TFA	36.64 ± 0.48	36.50 ± 0.50	0.240^a^
	3rd hour after TFA	36.67 ± 0.53	36.36 ± 0.48	0.013^a^
	4th hour after TFA	36.58 ± 0.55	36.50 ± 0.50	0.508^a^
	5th hour after TFA	36.56 ± 0.50	36.50 ± 0.50	0.642^a^
	6th hour after TFA	36.64 ± 0.48	36.36 ± 0.48	0.018^a^
Between group (F = 2.759, P = 0.101)^b^		F = 2.15, P = 0.14^b^	F = 1.09, P = 0.3^b^	
Systolic blood pressure, mmHg	Before TFA	142.08 ± 16.01	143.19 ± 17.10	0.777^a^
	Immediately after TFA	142.03 ± 14.23	141.36 ± 15.82	0.851^a^
	1st hour after TFA	146.64 ± 17.61	143.56 ± 18.73	0.474^a^
	2nd hour after TFA	142.44 ± 14.44	141.94 ± 16.23	0.891^a^
	3rd hour after TFA	142.31 ± 13.35	141.00 ± 18.06	0.729^a^
	4th hour after TFA	142.53 ± 12.56	143.19 ± 17.10	0.851^a^
	5th hour after TFA	140.86 ± 10.89	141.94 ± 16.23	0.741^a^
	6th hour after TFA	135.53 ± 17.21	143.56 ± 18.73	0.062^a^
Between group (F = 0.04, P = 0.84)^b^		F = 2.01, P = 0.11^b^	F = 1.16, P = 0.29^b^	
Diastolic blood pressure, mmHg	Before TFA	81.80 ± 6.34	81.10 ± 8.57	0.674^a^
	Immediately after TFA	80.00 ± 6.21	81.00 ± 8.26	0.574^a^
	1st hour after TFA	82.30 ± 14.73	81.40 ± 8.45	0.755^a^
	2nd hour after TFA	82.40 ± 5.69	79.70 ± 7.12	0.081^a^
	3rd hour after TFA	82.10 ± 5.15	81.10 ± 8.57	0.518^a^
	4th hour after TFA	80.50 ± 6.51	81.40 ± 8.45	0.586^a^
	5th hour after TFA	83.70 ± 5.44	79.70 ± 7.12	0.09^a^
	6th hour after TFA	82.20 ± 5.59	81.10 ± 8.57	0.507^a^
Between group (F = 0.60, P = 0.44)^b^		F = 0.61, P = 0.59^b^	F = 1.73, P = 1.96^b^	

*SD* standard deviation, *BPM* beats per minute, *TFA* transfemoral coronary angiography, *bpm* breaths per minute, *°C* degrees celsius, *mmHg* millimeter of mercury

^a^Independent t test

^b^RM-ANOVA

## Discussion

The current study demonstrated the effectiveness of changing a patients' position to decrease pain and vascular complications after TFA. Patients that were positioned with the head of bed significantly inclined and were supported into a semi-seated position reported less pain in the groin, leg, and back. Similar to previous researches, this study provides additional evidence that changing the position of patients after angiography reduced the severity of pain [18, 21, 23, 33]. This study additionally provided evidence that inclining the bed to 45° and supporting the patient into a semi-seated position was both safe and helpful in decreasing pain and improving physiological functioning. In the EG, back flexibility cause to relieve the tension level, which prevented an increase in the pain. Evidence showed that long-term immobilization imposes tension, and causes cellular ischemia and pain in the lumbar and the back [33, 34].

The current study demonstrated safety in changing the patient’s position to a semi-sitting position with angles of 15°–45° for up to 6 h after TFA as no patients experienced significant hematoma, hemorrhage, or urinary retention before or after the TFA. Other studies have reported similar results in terms of hematoma incidence [23, 34, 35], hemorrhage [34, 35], and urinary retention [2, 18, 21]. There were no bleeding complications because high-risk patients were not included in this study due to their risk of developing hematoma or hemorrhage. However, further evaluations on hematoma and hemorrhage in patients after TFA may be needed.

Similar to other research where the position following TFA was adjusted, this study showed that there was no significant difference between mean pulse rate, respiration rate, body temperature, or systolic and diastolic blood pressure in the two groups before and after TFA when the patient position was altered [10, 17, 26]. Evidence suggests that an increase in vital signs such as blood pressure and body temperature may indicate an increase in patients' cortisol levels due to increased pain [26]. Given the evidence in previous studies and the current study noting physiological stability and a decrease in pain scores for patients following TFA with altered positioning, it is strongly recommended that patients following TFA receive an inclined bed and semi seating positioning to improve post-TFA recovery.

### Limitations

Limitations of this study include variable pain thresholds for patients which could not be adequately measured, variable duration of angiography, and variable physician implemented pressure on the femoral access site after TFA. Also, the current study used the Christensen scale to measure the hematoma in the patients. The researchers suggest that more accurate methods such as Doppler ultrasound be used in future studies to assess hematoma.

## Conclusion

Overall, change in the position of patient (head of bed elevated from 15° to 45° and supported into semi-seated positioning) after TFA is an effective and safe procedure to reduce pain without changes to vital signs or complications such as hematoma, hemorrhage, and urinary retention that can cause to reduce the time of hospitalization. The current study showed that a simple, cost free, and safe nursing intervention can effectively improve the patients’ outcome after TFA. Further studies are recommended to investigate the possibility of increased patient movement directly after TFA to further support patient comfort and healing.

## Data Availability

The datasets used and/or analyzed during the current study are available from the corresponding author on reasonable request.

## Abbreviations

BMI: Body mass index
BPM: Beats per minute
bpm: Breaths per minute
CCU: Cardiac care unit
CG: Control group
CVD: Cardiovascular diseases
CAD: Coronary artery disease
°C: Degrees celsius
EG: Experimental group
ICU: Intensive care unit
INR: International normalized ratio
mmHg: Millimeter of mercury
PT: Prothrombin time
RM-ANOVA: Repeated measures analysis of variance
TFA: Transfemoral coronary angiography
VAS: Visual Analog Scale

## References

[1] Niveditha AS, Premavathy D (2019). Prevalence of heart disease among non-vegetarian individuals. Drug Invent Today.

[2] Bakhshi F, Namjou Z, Andishmand A, Panabadi A, Bagherinasab M, Sarebanhassanabadi M (2014). Effect of positioning on patient outcomes after coronary angiography: a single-blind randomized controlled trial. J Nurs Res.

[3] Gowshall M, Taylor-Robinson SD (2018). The increasing prevalence of non-communicable diseases in low-middle income countries: the view from Malawi. Int J Gen Med.

[4] Hatmi Z, Tahvildari S, Motlag AG, Kashani AS (2007). Prevalence of coronary artery disease risk factors in Iran: a population based survey. BMC Cardiovasc Disord.

[5] Sadeghi M, Haghdoost AA, Bahrampour A, Dehghani M (2017). Modeling the burden of cardiovascular diseases in Iran from 2005 to 2025: the impact of demographic changes. Iran J Public Health.

[6] Borren N, Maas AH, Ottervanger JP (2015). Stop invasive coronary angiography as the gold standard for the diagnosis of stable angina!. Interv Cardiol.

[7] Tavakol M, Ashraf S, Brener SJ (2012). Risks and complications of coronary angiography: a comprehensive review. Glob J Health Sci.

[8] Manda YR, Baradhi KM. Cardiac catheterization, risks and complications. 2018. https://www.ncbi.nlm.nih.gov/books/NBK531461/. Last Update: June 22, 2020. 30285356

[9] Kurt Y, Kaşıkçı M (2019). The effect of the application of cold on hematoma, ecchymosis, and pain at the catheter site in patients undergoing percutaneous coronary intervention. Int J Nurs Sci.

[10] Kardan M, Zarei B, BahramiTaghanaki H, Vagharseyyedin SA, Azdaki N (2020). The effects of foot reflexology on back pain after coronary angiography: a randomized controlled trial. Complement Ther Clin Pract.

[11] Rani S, Lakshmi R, Pillai A, Nisha S (2016). The risk factors associated with complications of coronary angiogram: a cross-sectional observational study. Int J Adv Med Health Res.

[12] Sulzbach-Hoke LM, Ratcliffe SJ, Kimmel SE, Kolansky DM, Polomano R (2010). Predictors of complications following sheath removal with percutaneous coronary intervention. J Cardiovasc Nurs.

[13] Anjum I, Khan MA, Aadil M, Faraz A, Farooqui M, Hashmi A (2017). Transradial vs. transfemoral approach in cardiac catheterization: a literature review. Cureus.

[14] Kim K, Won S, Kim J, Lee E, Kim K, Park S (2013). Meta-analysis of complication as a risk factor for early ambulation after percutaneous coronary intervention. Eur J Cardiovasc Nurs.

[15] Fereidouni Z, Morandini MK, Kalyani MN (2019). The efficacy of interventions for back pain in patients after transfemoral coronary angiography: a rapid systematic review. J Vasc Nurs.

[16] Hajbaghery MA, Moradi T, Mohseni R (2014). Effects of a multimodal preparation package on vital signs of patients waiting for coronary angiography. Nurs Midwifery Stud.

[17] Mohammady M, Atoof F, Sari AA, Zolfaghari M (2014). Bed rest duration after sheath removal following percutaneous coronary interventions: a systematic review and meta-analysis. J Clin Nurs.

[18] Abdollahi AA, Mehranfard S, Behnampour N, Kordnejad AM (2015). Effect of positioning and early ambulation on coronary angiography complications: a randomized clinical trial. J Caring Sci..

[19] Naseri Salahshour V, Sabzali Gol M, Basaampour SS, Varaei S, Sajadi M, Mehran A (2017). The effect of body position and early ambulation on comfort, bleeding, and ecchymosis after diagnostic cardiac catheterization. J Client-Centered Nurs Care..

[20] Krouscoup R (2000). Nursing interventions to decrease bleeding at the femoral access site after percutaneous coronary intervention. Am J Crit Care.

[21] Neishabouri M, Haghighi N, Gilvari T, Haghighat S (2020). Effect of changing position and early mobilization on back pain and vascular side effects in patients after coronary angiography. J Nurs Midwifery Sci.

[22] Rezaei-Adaryani M, Ahmadi F, Asghari-Jafarabadi M (2009). The effect of changing position and early ambulation after cardiac catheterization on patients’ outcomes: a single-blind randomized controlled trial. Int J Nurs Stud.

[23] Valiee S, Fathi M, Hadizade N, Roshani D, Mahmoodi P (2016). Evaluation of feasibility and safety of changing body position after transfemoral angiography: a randomized clinical trial. J Vasc Nurs.

[24] Pool J, Dercher M, Hanson B, Heiman L, Li Y, Schraeder K, Schultz MP, Ziglinski S, Ebberts M (2015). The effect of head of bed elevation on patient comfort after angiography. J Cardiovasc Nurs.

[25] Sedighi F, Barkhordari Sharifabad M, Nasiriani K, Fallahzadeh H (2018). The effect of bed angle on back pain, urinary retention and vascular complications after coronary angiography. Iran J Cardiovasc Nurs.

[26] Heravi MAY, Yaghubi M, Joharinia S (2015). Effect of change in patient's bed angles on pain after coronary angiography according to vital signals. J Res Med Sci.

[27] Nasiri M, Farsi Z (2017). Effect of light pressure stroking massage with sesame (*Sesamum indicum* L.) oil on alleviating acute traumatic limbs pain: a triple-blind controlled trial in emergency department. Complement Ther Med..

[28] von Baeyer CL, Spagrud LJ (2007). Systematic review of observational (behavioral) measures of pain for children and adolescents aged 3 to 18 years. Pain.

[29] Narayan MC (2010). Culture's effects on pain assessment and management. Am J Nurs.

[30] Dowlatshahi D, Demchuk A, Flaherty M, Ali M, Lyden P, Smith E (2011). Defining hematoma expansion in intracerebral hemorrhage: relationship with patient outcomes. Neurology.

[31] Delgado DA, Lambert BS, Boutris N, McCulloch PC, Robbins AB, Moreno MR, Harris JD (2018). Validation of digital visual analog scale pain scoring with a traditional paper-based visual analog scale in adults. JAAOS..

[32] Chair SY, Thompson DR, Li SK (2007). The effect of ambulation after cardiac catheterization on patient outcomes. J Clin Nurs.

[33] Cha NH, Sok S (2016). Effects of position change on lumbar pain and discomfort of Korean patients after invasive percutaneous coronary intervention: a RCT study. J Phys Ther Sci.

[34] Rai P, Bagga S, Gopichandran L, Sharma YP (2019). A randomized controlled trial on position change followed by early ambulation after trans-femoral coronary angiography. Asian J Nurs Edu Res..

[35] Yeganekhah M, Tehrani D, Ziyuayinejad M (2012). Comparing different ways of position on vascular complications after coronary angiography: a randomized clinical trial. Qom Univ Med Sci J..

